# Providing Quality of Care in Fragile and Vulnerable Settings: Lessons from South Sudan

**DOI:** 10.5334/aogh.3506

**Published:** 2021-12-22

**Authors:** Kevin Gianaris, Jacob Atem, Allison P. Chen, Alexander H. Chang, Anna Russell, Edbert B. Hsu

**Affiliations:** 1 Southern Sudan Healthcare Organization (SSHCO), Juba, Central Equatoria State, South Sudan, US; 2 Department of Surgery, Johns Hopkins University, Baltimore, MD, US; 3 Center for Humanitarian Health, Johns Hopkins Bloomberg School of Public Health, Baltimore, MD, US; 4 Department of Biology, Johns Hopkins University, Baltimore, MD, US; 5 Lewis Katz School of Medicine at Temple University, Philadelphia, PA, US; 6 Department of Emergency Medicine, Johns Hopkins University, Baltimore, MD, US; 7 Center for Global Emergency Care, Johns Hopkins University, Baltimore, MD, US

## Abstract

**Background:**

In 2020, the World Health Organization (WHO) released a report concerning planning and actions to provide quality of care in fragile, conflict-affected, and vulnerable areas. South Sudan, the world’s newest country, has encountered both natural and man-made disasters in recent years that have posed marked challenges to delivery of care. The Southern Sudan Healthcare Organization (SSHCO) operates as a non-governmental organization (NGO) in this setting, delivering and improving healthcare through war, flooding, and infectious outbreaks.

**Objective:**

The goal of this paper is to highlight the challenges faced in providing care in South Sudan from an NGO perspective and apply the recent WHO guidelines on quality of care to optimize practical implementation.

**Method:**

Each of the WHO’s eight elements for quality of care in South Sudan were examined in relation to the experience of SSHCO from 2013–2021. Analysis included: 1. summary of the WHO element; 2. examples of successful implementation; 3. barriers to implementation; and 4. recommendations to improve implementation.

**Findings:**

The team found that communication and coordination were the most important aspects of improving quality of care in South Sudan. These should be prioritized and include intergovernmental partners, the local and national Ministry of Health (MOH), NGOs, and community stakeholders. Communication and coordination should foster community engagement, improved data collecting and reporting, and sharing of publicly accessible information. Better clinical staff training and governance are also required to ensure the most effective use of limited resources.

**Conclusion:**

South Sudan faces many barriers to quality of care with communication and coordination identified among the foremost issues. Practical application of the WHO elements of quality of care can assist NGOs in effectively identifying areas for improvement to deliver better quality essential health services.

## Introduction

In 2020, the WHO released a document outlining plans and practical actions to be taken in providing quality care in fragile, conflict-affected, and vulnerable settings [1] The document outlines eight essential elements for strategic planning and action to provide quality of care in these contexts. These elements are:

1. *Service priorities and quality goals*,
2. *Shared local understanding of quality*,
3. *Stakeholder mapping and engagement*,
4. *Situational analysis – state of quality*,
5. *Governance for quality*,
6. *Interventions for quality improvement*,
7. *Health information systems and quality assessment*, and
8. *Quality measurement* (***Table 1***).

**Table 1:** Summary of elements from WHO quality of care in fragile, conflict-affected, and vulnerable settings: tools and resources compendium.


ELEMENTS	DESCRIPTIONS

Service priorities and quality goals	Coordinating with existing health systems to provide quality care that addresses the most pertinent needs of the population

Shared local understanding of quality	Engaging with the community to ensure that the work is relevant and in sync with local goals

Stakeholder mapping and engagement	Collaborating with stakeholders to promote quality and responsiveness, among other benefits

Situational analysis-state of quality	Performing assessments of needs, capabilities, and quality

Governance for quality	Ensuring oversight and governance in supporting quality work

Interventions for quality improvement	Improving quality through actionable plans and timely delivery

Health information systems and quality assessment	Promoting decision-making based on assessment and data driven solutions

Quality Measurement	Measuring quality through valid indicators adapted to local context


**Figure 1:**
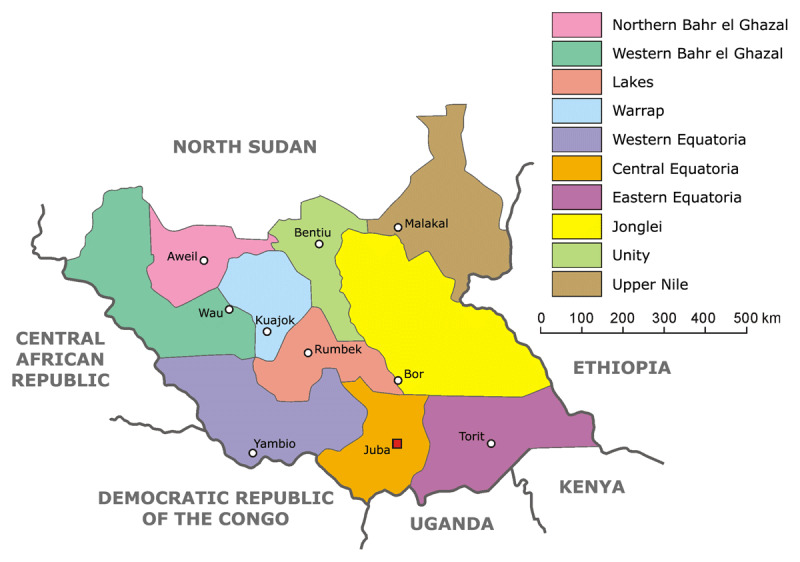
Map of South Sudan [16].

Representing one of the most fragile, conflict-affected, and vulnerable nations, the Republic of South Sudan, established in 2011, is the world’s newest country. Wracked by civil war and natural disasters, the nascent nation has faced significant ongoing challenges surrounding food security and violence [2] (***Figure 1***). As of mid-2020, there are over 2.2 million South Sudanese refugees as well as nearly 1.5 million internally displaced persons (IDPs) in South Sudan [3]. In particular, the Jonglei state has experienced major workforce shortages, precipitous declines in agricultural production during the conflicts, and extremely poor health indicators [4,5]. In August 2019, 1.25 million people in the Jonglei state faced severe acute food insecurity, at a level of crisis, emergency, or famine level conditions (Integrated Food Security Phase Classification [IPC] Phases 3, 4, or 5). According to this classification, these are the highest numbers for any region in South Sudan after taking into account active humanitarian assistance [6]. Although a new government was formed in February 2020, humanitarian crisis conditions persist and by 2021, six counties have now been identified as food insecure [7].

Delivery and provision of healthcare have been directly impacted by a shortage of personnel, facilities, and resources and are continuously disrupted by conflict. In 2011, there was roughly one trained physician for every 65,000 individuals, and one midwife for every 40,000 [8]. Clinics and other locations providing health services, including teaching hospitals, have been attacked throughout the fighting, and in the Jonglei State, over 50 percent of health facilities are non-functional, among the highest rates in the country [9,10,11]. International aid groups like the International Committee of the Red Cross (ICRC), Medecins Sans Frontieres (MSF), and local non-governmental organizations (NGOs) have been significant providers and funders of healthcare in South Sudan since 2011, with NGOs accounting for 70 percent of health services in 2015 [12,13].

The Southern Sudan Healthcare Organization (SSHCO) is a U.S.-based NGO that built and operates the Maar Primary Health Care Center (MPHCC) in the town of Maar in the Twic East County, Jonglei State (6°54’17.0”N 31°21’15.3”E) with the stated mission of “Bringing Health and Hope to Where it is Lost.” The clinic primarily treats patients from the Duk, Bor, and Twic East counties, with an estimated catchment population of approximately 560,000, including 122,320 from Duk, 315,671 from Bor, and 122,008 from Twic East [14]. The clinic also serves communities along the Nile River which are not accessible by car and some patients walk over a day’s journey to access the clinic services. Complicating access is the presence of the Sudd wetland, which surrounds the White Nile River at the western border of Jonglei state. During the dry season, it is difficult terrain, and during the wet season, from April to November, the swamp regions of the Sudd can more than double in size flooding the roads [15]. Primary services provided include vaccinations, medication distribution, family planning services, maternity care, and infectious disease treatment. Local healthcare workers provide malnutrition assistance and a referral system to larger hospitals. There were 22 full-time staff employed at the clinic including clinical officers, registered nurses, a pharmacist, a lab tech, community health workers, cleaning staff, and security guards. Fifteen hospital beds allowed for limited inpatient treatment, although most services provided were on an outpatient basis. The clinic saw an average of 4,000–4,500 patients per year during continual operations from 2013–2015. Selected cases were referred to the hospital in Bor or the Panyigoor County Hospital in Twic East County, with the most complex cases going to the capital city, Juba. Since 2020, clinical teams have also provided care to the local and IDP populations through mobile clinics in Mangalla in Juba County in the Central Equatoria State (CES), seeing between 1,200–2,000 patients per month. SSHCO employs 17 clinical staff operating mobile clinics at the Mangalla Humanitarian Site.

This report relies upon the experience of SSHCO in the context of a fragile, conflict-affected and vulnerable setting to apply the eight essential elements of action planning laid out by the WHO. Practical considerations and challenges in administering care in South Sudan as well as areas for improvement and examples of successful operations are discussed.

## Methods

The research team systematically examined each of the WHO’s eight elements for quality care and applied them to the experience of SSHCO as an NGO and as a member of the Health Cluster from 2013 to 2021. This paper will examine both sites where SSHCO has been operating: MPHCC and Mangalla Humanitarian Site. Analysis of these elements included: 1. summary of the WHO element; 2. examples of successful implementation; 3. barriers to implementation; and 4. recommendations to improve implementation.

## Analysis

### 1. Service priorities and quality goals

This element describes the need for health actors to coordinate with existing health systems in providing quality care that addresses the most pertinent needs of the population. It focuses strongly on following the lead of local health services to ensure directed, effective care. Understanding existing local priorities enables partners to align their own goals to promote efficient efforts and integrate political and financial support.

In South Sudan, local goals have been outlined by the national Ministry of Health (MOH) and by international organizations. The United Nations Office for the Coordination of Humanitarian Affairs (UNOCHA) compiled an inter-agency Humanitarian Response Plan (HRP) based on a Humanitarian Needs Overview created that year [17]. The HRP is developed from the data, experiences, and input of various partners in the country and is meant to delineate a directed plan of action for all partners to work toward; it is not intended to be a comprehensive understanding of every issue affecting the country. The MOH also releases National Health Policy reports, with the latest one addressing the years 2016–2026 [18]. These identify the most prominent healthcare needs and objectives and outline national public health priorities.

In addition to long-term planning, immediate needs must be addressed. During particular times, certain diseases are far more common and present more danger to the communities in South Sudan than others. Thus, prioritization of health services is necessary. For example, SSHCO staff have observed regular increases in malaria and acute watery diarrhea cases during the rainy season and outbreaks such as cholera require immediate attention. Priorities should be agreed upon by the in-country health partners through timely and accurate data and on-the-ground reporting of changing needs. As another example, SSHCO noticed a marked uptick in acute watery diarrhea cases at their mobile clinics due to extremely poor water, sanitation, and hygiene (WASH) conditions at the Mangalla Humanitarian Site. This alert was raised in the WHO’s Early Warning, Alert, and Response System (EWARS) leading to investigation of cases and testing of samples. In this situation, resources were diverted from other rural areas to address this at the Humanitarian Site, informed by data and made possible through the participation of many stakeholders like the Surveillance Officer from CES Ministry of Health, National Ministry of Health, Health Cluster, and WHO.

The development of successful plans is closely tied to widespread participation and accurate flow of information. Where this fails is in the reporting of gaps, with weekly reporting to the Integrated Disease Surveillance Reporting (IDSR) system consistently below 80% [19]. The constant political and social changes in South Sudan also impose difficulties. Specialized training for clinical staff is needed to ensure timely and accurate technical reporting of data. For up-to-date information, an open direct line of communication with the South Sudan Health Cluster partners, such as via a closed monitored online forum, could allow partners to directly share pertinent information. Though there is room for improvement, this element is now being accomplished relatively effectively in South Sudan.

### 2. Shared local understanding of quality

The WHO stresses providing effective, safe, people-centered, timely, equitable, integrated, and efficient care with a shared understanding of quality with the local population. They advocate for the use of stakeholder engagement to create an understanding of how interventions may benefit the local communities in line with local priorities. While these aspects may be difficult to incorporate in fragile, conflict-affected, and vulnerable settings, developing shared local understanding of quality is nevertheless important for an effective action plan.

In South Sudan, this element is particularly challenging, given the insular nature of many communities and constant population flux due to disasters and conflict. The element can be addressed with processes that engage local stakeholders to incorporate their input through interviews and focus groups. Often, this is incorporated within needs assessments conducted before projects are initiated in a region.

In addition, African-run NGOs such as SSHCO benefit strongly from durable relationships with the community built over many years. Together with leadership from members of the community, local culture is better understood and facilitates dialogue. The Village Health Committees convened by SSHCO enabled local partners in Maar to gather to voice concerns and complaints. This participation helped SSHCO design care that incorporates local input and leads to a shared understanding of quality care. An example of this was during the COVID-19 vaccination campaign where the SSHCO gathered the chiefs and key stakeholders in the community to discuss the risks and benefits of the COVID-19 vaccine being brought to their community. The SSHCO team provided accurate information, answered questions, and dispelled rumors before beginning the campaign. Furthermore, local NGOs can ensure continuity and consistency of care before, during and after disasters. There has been a recent international push by African leaders to favor decolonized aid by funding local and regional based groups in Africa [20]. To improve this element, empowerment of local NGOs and government organizations through funding and administrative support can create a stronger local understanding of quality.

### 3. Stakeholder mapping and engagement

This element describes the process of coordinating action among stakeholders to promote quality and responsiveness, among other benefits. In South Sudan, this is most directly accomplished through the Cluster system set-up by the WHO and requested by the government of South Sudan. The state, non-state, and intergovernmental health partners in the country are convened twice per month. The Health Cluster (HC) also serves as the chief source of up-to-date information on diseases and other public health related issues in South Sudan. It is essential that the Health Cluster and the associated NGOs coordinate with and support the MOH mission rather than duplicating or undermining efforts.

As an NGO, SSHCO maintains an independent relationship with the County Health Department (CHD), State Ministry of Health (SMOH) and the National Ministry of Health (MOH). The MOH serves as the most robust coordinating body that can provide resources effectively when engaged correctly. SSHCO maintains communication and partners with the MOH on the county, state, and national level to complement the local healthcare system. Both offer and receive resources that are best suited for each respective partner. Ensuring accountability from the MOH is necessary for partnership. The Health Cluster system successfully accomplishes this by readily including the National MOH, SMOH, CHD and allowing new partners to enter with few barriers. The Cluster coordinator is required to engage all pertinent partners efficiently and ethically. However, in some cases, the amount of coordination or communication needed is more than the Cluster coordinator. Allowing a degree of outside coordination beyond the health Cluster may ease some of the burden placed on the Cluster coordinator.

### 4. Situational analysis – state of quality

The WHO affirms that organizations can observe the state of quality by assessing the current level of needs of organizations, understanding where improvements can be made, recognizing where needs and challenges lie and identifying successes to emulate. A situational analysis is best accomplished by conducting on-the-ground observation and engaging stakeholders to thoroughly understand the impacts of care. Desk review, stakeholder interviews, observation, stakeholder review and validation, and review of challenges common in fragile, conflict-affected, and vulnerable settings, have all been outlined as methods of performing this analysis [1].

In South Sudan, it is necessary to understand the barriers to care, infrastructure successes and failures, the political, social, and cultural climate, and local capacity. Analyses take different forms, with rapid needs assessments indicated in crisis situations and more in-depth annual reports required for long-term efforts at established facilities. Reporting should be sufficiently comprehensive to demonstrate the strengths, weaknesses, opportunities, and threats present. As an example, risk analysis is particularly relevant for South Sudan to develop appropriate mitigation strategies. The SSHCO administrative team recently performed a comprehensive risk analysis in Mangalla after interviewing their clinical staff and visiting different parts of the camp. The SSHCO risk analysis included security risks, risks to beneficiaries, quality of clinical care, and potential spending waste with proposed mitigation strategies for each issue. A “state of quality” report will reflect the environment within which the healthcare organizations operate and can be compared to other partners in the region for contextualization. This type of assessment is best done where it can be confirmed or validated by other independent partners.

For data to be comparable, organizations must communicate about topics of collection, methods, classification, and organization of collected data. In addition, it is important that partners coordinate to avoid duplicative efforts. The Cluster must play a crucial role in disseminating the most up-to-date information to the health partners for situational awareness and coordination including information about attacks on healthcare workers and developing security threats.

### 5. Governance for quality

Lack of governance is often a key issue in fragile, conflict-affected, and vulnerable settings. Good governance can provide accountability and effective collaboration to promote quality care. A recognized governing body can designate roles and responsibilities as well as monitor care delivery.

Despite facing some of the most extreme health and nutrition risks, South Sudan suffers from severe underfunding. This makes governance for quality paramount to achieve success in humanitarian crisis response. Avoiding duplication of resources and resource waste has been both proven and necessary in low-income countries suffering from conflict [21]. Governance for quality is mainly performed by the Health Cluster leadership and MOH in South Sudan, but health partners can also communicate independently to ensure the most efficient use of resources. The MOH is meant to regulate standards of care, yet the Cluster has a strong influence in these regulations by directing a substantial portion of available funding.

NGOs need to be aware of the governance landscape and collaborate as effectively as they can within it. Often in conflict-affected settings, information may lag behind necessary action and health partners must activate contingency plans before an issue is well recognized or fully understood by the governing body. This was the case when SSHCO was forced to evacuate its staff during a state conflict in 2013. An evacuation to a relatively safer area of South Sudan came with costs and left a gap for healthcare services. Aid workers remain a target for violence – this increased threat limits the capabilities of health facilities and can even lead to their closure as is the case of the PHCC in Mangalla. A total of 128 humanitarians have lost their lives since 2013 with five killed thus far in 2021 [22]. To improve this governance, there must be transparency with partners and accountability. Partners should certainly work with governance but also understand its many limitations in low resource settings and adjust activities accordingly.

### 6. Interventions for quality improvement

The success of quality improvement will rely on implementation of actionable plans. The WHO outlines five areas to target for quality improvement: 1) ensure access and basic infrastructure, 2) shape the system environment, 3) reduce harm, 4) improve clinical care, and 5) engage patients, families, and communities. Mapping the current quality standards, developing clear implementable interventions, and creating a plan to implement those interventions to improve care have been proposed.

Quality improvement is particularly difficult to achieve in South Sudan, where the level of clinical training is one of the lowest in the world. Moreover, the workforce faces a lack of investment in human resources. In South Sudan, quality can be most directly improved through increased training of clinical staff and increased systems supporting their care. Developing systems of support should be directly informed by the clinical staff on the ground coupled with input coming from the beneficiaries. The best place to begin is an understanding of the current local or regional circumstances. Communication with other partners in the area about successes and failures is also crucial to developing models that work. Having a strictly human resources policy for an organization can most effectively eliminate nepotism, favoritism, and overall corruption in the hiring process.

In South Sudan, the SSHCO finds its biggest challenges in the areas of improving clinical care and adequately training its staff. Working in the extremely remote region of Maar in Twic East County renders accessibility to training difficult. Costs for training are also extremely high. To adapt to this, dedicated trainers have traveled to Maar and implemented a “train the trainer model” where more highly trained staff can instruct others in basic techniques. Incorporating report findings from other NGOs about their successful methods can also help inform these practices.

Taking these into account, SSHCO defines the interventions and sets realistic timelines to successfully implement these goals. For broad-based country level improvement, more consistent training held by the Health Cluster and improved sharing of successes are needed.

### 7. Health information systems and quality assessment

Health information systems mainly provide data that can drive improved quality care. Following the goal of data-driven solutions, health information has grown increasingly powerful in supporting quality of care. During many conflicts, these systems are disrupted, if not stalled, which can lead to discord and weakens decision making. The WHO suggests a comprehensive mapping of the current health information system followed by implementation of health system improvement plans to strengthen the system.

The Cluster uses EWARS to provide IDSR data on a weekly basis from all the health partners’ facilities. Partners can automatically raise alerts about local disease outbreaks and activate partner responses. While several partners have acquired satellite internet and Thuraya phones to ensure stable communications and reporting, this is very expensive, with calls to Maar costing upwards of $5 per minute. Health information systems often rely on reporting based upon internet and cellular service. Some innovative solutions such as the Kobo toolbox have been invented to improve health information, and the SSHCO has recently adopted its use in Mangalla. It can be used to record information offline and send it when internet service is available. For reporting purposes in rural South Sudan, organizations may need to augment their information-reporting systems with paper forms that can be sent either physically or by text or WhatsApp to administrators who can enter the information electronically. WhatsApp and phone calls remain SSHCO’s most direct method of obtaining up-to-date information from the field in the case of emergency or urgent needs.

Apart from this, rapid needs assessments, gender and disability analyses, household surveys, and interviews as well as community observations help inform the planning and response during conflict and disaster. Progress in health information systems faces many challenges, but increased funding for communication tools, such as satellite internet, and additional technical staff training are concrete ways to improve quality care.

### 8. Quality measurement

Quality must be measured and can most usefully be accomplished through simple, practical, validated indicators. It is important not to add extraneous information demands or complex metrics that may derail reporting efforts in low-resource settings. The WHO outlines key methods of surveying existing indicators, reviewing the indicator strengths and weaknesses, and selecting the most suitable practical indicators for these contexts.

In South Sudan, the Cluster uses a set of indicators that target key diseases, including malaria, acute watery diarrhea, acute bloody diarrhea, and respiratory tract infections. Certain external indicators may reflect broader metrics, such as the availability of obstetric surgical care as a proxy for the availability of essential surgical care at the facility. Other metrics might be informed by social disparities including gender, disability, or special needs issues, ensuring a more balanced approach for achieving sustainable development goals.

Many areas of South Sudan do not have internet service or adequately trained IT staff. Accordingly, measurement tools should be aimed toward the quality of data obtained, rather than the quantity. With increasing consistency and familiarity, additional important and relevant disaggregates and indicators for data collection may be gradually implemented. Monitoring and evaluation are key to ensuring continuous quality improvement amidst the ebb and flow of conflicts and disasters.

## Discussion and Recommendations

While the WHO Quality of Care framework provides preliminary guidance for a team such as SSHCO’s to assess healthcare quality, several aspects of quality care remain unaddressed. First, the sustainability of care, especially in the treatment of chronic diseases, is largely not captured by the framework. Rates of chronic disease, including HIV, are increasing in fragile settings [23]. These diseases require prolonged care and case management which can only come through sustainability of services and collaboration between government and non-governmental partners. Without an emphasis on long-term planning for both systems and patients, patient adherence and retention will be difficult to be maintained. When a healthcare system is largely dependent on non-governmental organization support, sustainability of services for individuals must be at the forefront of planning.

Secondly, three of the eight quality elements focus on measurement of quality (situational analysis, health information systems and quality assessment, and quality measurement). While measuring quality is undoubtedly important to understanding the baseline situation and tracking progress, the emphasis on measurement may place undue burden on an already weak healthcare system. Much of the data needed to be collected would need some involvement from already taxed healthcare workers and administrative staff. The quality of care should be measured but organizations and governments should be cognizant of the burden of the data collection, management, analysis, and reporting in requiring extensive measures to be collected.

Recommendations follow the eight elements of WHO’s Quality of Care framework (***Table 2***). First, to improve service priorities and quality goals, the Health Cluster coordinator and WHO can perform a situational analysis to determine barriers preventing the Cluster system and Cluster coordination from working effectively within the South Sudan context. This could include issues that are unique to South Sudan as well as barriers that are representative of the Cluster system at large. In addition, there is currently a closed monitored online forum shared by all Cluster members. At the present, this is most often used to post job openings but can be expanded to information sharing between Cluster members.

**Table 2:** Recommendations for Implementation of WHO Quality Care Elements.


ELEMENT	AUDIENCE	ACTION ITEMS

Service priorities and quality goals	Cluster coordinator	Undertake situational analysis to determine barriers to prioritization and coordination among all stakeholders

	Cluster Coordinator	Expand usage of closed monitored online forum to increase coordination among Cluster members

Shared local understanding of quality	All NGOs	Increase local leader and community participation

	All NGOs, led by Cluster coordinator	Improve robustness of existing equity analyses to determine which voices are being heard at stakeholder meetings and how to create more diverse program advisory committees

	Donors	Conduct assessment on regulations or policies that may be prohibitive to African-run NGOs applying for and receiving grants

	Donors	Reduce administrative burden on grantees

	Donors	Provide grant application-writing workshops to African-run NGOs

Stakeholder mapping and engagement	SSHCO	Create stakeholder map and methods of engagement matrix

	Cluster coordinator	Empower MOH to have larger leadership role within Cluster

Situational analysis-state of quality	Cluster coordinator	Determine current overlap in programs and geographic regions between Cluster organizations and produce accompanying report

Governance for quality	All NGOs	Support MOH through leadership training

	International NGOs/Donors	Encourage international NGOs to adopt a health-systems strengthening approach, working in partnership with the MOH

Interventions for quality improvement	All NGOs	Provide scholarships for future healthcare workers

	All NGOs	Target capable local staff at all levels and fund continuing education

Health information systems and quality assessment	SSHCO	Create specialized training for data clerks to ensure timely reporting of IDSR data

Quality measurement	SSHCO	Create and improve quality measurement indicators such as healthcare staffing levels, pharmaceutical access, and procedure usage and access


To improve shared local understanding of quality (WHO Element 2), both international and local NGOs should increase local leader and community participation in programs. This participation should take place before, during, and after program development and implementation. While equity analyses are already in place in the HRP, expanding the robustness of these analyses would improve the ability for women, persons with disabilities and older persons to participate in community decision-making. Donors also play a large role in increasing local participation and improving local NGOs’ ability to receive funding. Donors can conduct an assessment of their own regulations that are either directly or indirectly prohibitive to successful application by local NGOs. Using these assessments, donors can also opt to lessen administrative burden on grantees, which unfairly penalizes smaller local NGOs. Lastly, donors can provide grant application-writing workshops for local NGOs to gain access to knowledge and skills to compete successfully for grants.

A key part of improving local understanding is engaging in stakeholder mapping and developing relationships with stakeholders. SSHCO should undertake a stakeholder mapping matrix exercise to determine how best to interact with the MOH, the Cluster, local NGOs and international NGOs to increase opportunities for collaboration. In addition, empowering the MOH to undertake a leadership role within the Cluster would have sustainable impacts on centering South Sudanese leadership.

A common issue within international development is multiple NGOs working in the same area, duplicating programmatic and geographic efforts. One step towards a longer-term solution would be the development of a report focused on the areas within South Sudan that currently have NGO duplication of efforts. If done by the Cluster coordinator, this could be shared with Cluster members.

Element 5 of WHO’s framework pertains to governance for quality. The Cluster is currently the lead healthcare entity in South Sudan, but efforts should be made to empower the MOH to take a stronger, more authoritative role in both the Cluster and in negotiating work done by NGOs. This can be done through leadership training at the MOH and through donors requiring or encouraging international NGOs to focus on health systems strengthening within the MOH, rather than building parallel health systems outside of the national health system. The sixth element is interventions for quality improvement. The largest gap within this element is the small number of trained local healthcare workers within South Sudan. This is both a training and a workforce problem. NGOs should undertake training of current healthcare staff and provide continuing education in addition to providing scholarships for future healthcare workers. Importantly, the education should take place within existing schools and in cooperation with the MOH when possible.

Data systems in South Sudan are weak and often have low data quality and quantity. Even one of the most critical data systems, the IDSR, has low completeness from the NGOs. Thus, SSHCO and other NGOs should focus on creating specialized training for data clerks to ensure reporting of IDSR data in a timely and complete fashion. Lastly, quality measurement needs to be undertaken as it is currently not a large focus in South Sudan. By using pre-existing tools developed by WHO and others, SSHCO can create and improve their own tools to begin measuring care provided by local healthcare workers.

## Conclusion

Achieving quality of care in fragile, conflict-affected, and vulnerable settings principally requires coordination and communication. The South Sudan Health Cluster facilitates strong collaboration among health partners. However, the coordination through the Cluster is only as effective as its leadership and participation from stakeholders. Beyond this, it is the responsibility of partners to collaborate with County Health Departments, State Ministries of Health, the National Ministry of Health, and other NGOs in South Sudan to increase accountability and promote equitable resource allocation. External health partners in South Sudan should aim not to replace the local health efforts but rather to focus on complementing existing structures with a goal of long-term sustainability.
